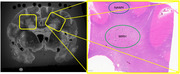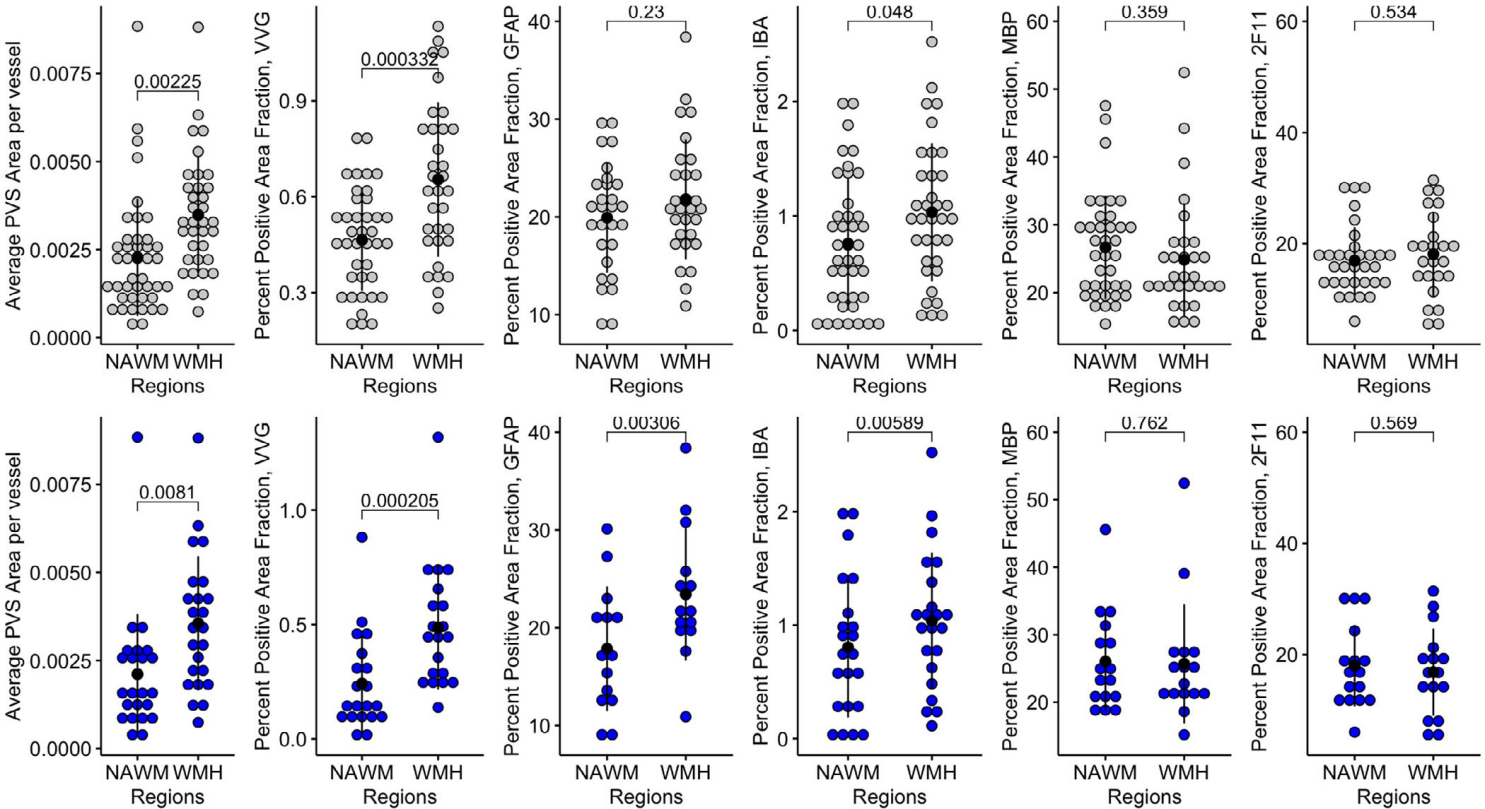# Multiplexed pathological markers of white matter hyperintensities show greater vascular and inflammatory injury than neuronal damage

**DOI:** 10.1002/alz70856_102482

**Published:** 2025-12-25

**Authors:** Swati Rane Levendovszky, Joshua C Russell, Christine L Mac Donald, C. Dirk Keene

**Affiliations:** ^1^ University of Kansas Medical Center, Kansas City, KS, USA; ^2^ University of Washington, Seattle, WA, USA; ^3^ Department of Pathology, University of Washington, Seattle, WA, USA

## Abstract

**Background:**

White matter hyperintensities (WMHs) are radiographic features on T2‐MRI that provide no information about underlying pathology. Neuropathological studies show that there are multiple pathological substrates such as neurodegenerative, vascular, and neuroinflammatory underlying WMHs.

**Method:**

Fifty‐two MRI‐matched tissue blocks were identified containing one or all WMHs and normal‐appearing white (NAWM) and gray matter tissue types. All samples and processes were standardized to ensure consistency. Staining was performed on Leica BOND‐MAX or BOND‐RX autostainers. We assessed small vessel pathology, including hyalinization, arteriolosclerosis, and disruptive processes (Smooth Muscle Actin, SMA), axon loss and demyelination (Eosin/LFB, and myelin basic protein or MBP), neurofilament light chain protein degradation (2F11), and gliosis (IBA). Following whole slice brightfield microscopy and image capture, a supervised semi‐automated quantification was conducted using HALO. NAWM and WMH regions were drawn using the FLAIR MRIs as reference on the LFB images and applied across all stains. For SMA, the total area stained was divided by number of perivascular spaces to obtain average perivascular space size. Area fraction (area stained/total area of the region) was calculated for the rest. All measures were compared between the two regions. Since cellular and vascular architecture of tissue could differ based on sampled location and affect interpretation, we conducted a paired analyses comparing the two regions of interest in the same tissue block.

**Result:**

Figure 1 shows the MRI block showing tissue location and the corresponding Eosin/LFB block with NAWM and WMH regions. PVS sizes, collagenosis, and gliosis were significantly greater in WMH than NAWM in both the unpaired and paired analyses (Figure 2). Astrocytic injury was only significantly different in the paired analyses. Markers of myelination or neurofilament light chain were not significantly different between NAWM and WMH.

**Conclusion:**

Vascular and glial pathology is most common in WMHs. Given that the astrocytic marker was only significant in the paired analysis, it may represent a more localized pathology. We are unsure why no neuronal pathology markers were identified in the WMH. This could mean, that neuronal injury is not as severe in WMH or there is widespread aging‐related diffuse white matter injury that overshadows the WMH pathology.